# Alignment between timing of ‘highest caloric intake’ and chronotype in relation to body composition during adolescence: the DONALD Study

**DOI:** 10.1007/s00394-023-03259-w

**Published:** 2023-10-20

**Authors:** Nicole Jankovic, Sarah Schmitting, Bianca Stutz, Bettina Krüger, Anette Buyken, Ute Alexy

**Affiliations:** 1 Nutritional Epidemiology, Department of Nutrition and Food Sciences, DONALD Study, Rheinische Friedrich-Wilhelms-University Bonn, Heinstück 11, 44225 Dortmund, Germany; 2 Faculty of Natural Sciences, Institute of Nutrition, Consumption and Health, Paderborn University, Warburger Str. 100, 33098 Paderborn, Germany

**Keywords:** Adolescence, Body composition, Chronotype, Prospective cohort study, Diet

## Abstract

**Purpose:**

Our aim was to assess alignment in timing of ‘highest caloric intake’ with individual chronotype and its association with body composition in adolescents.

**Methods:**

We used repeatedly collected data from *n* = 196 adolescents (age 9–16 years, providing *N* = 401 yearly questionnaires) of the DONALD open cohort study. Chronotype was assessed by the Munich Chronotype Questionnaire from which midpoint of sleep (MSFsc) was derived. A sex- and age-specific diet-chrono-alignment score (DCAS) was calculated as the difference in hours between the chronotype-specific median timing of highest caloric intake of the studied population and the individual timing of ‘highest caloric intake’ or vice versa. Repeated-measures regression models were applied to study cross-sectional and longitudinal associations between the DCAS and body composition, e.g., Fat Mass Index (FMI) or Fat Free Mass Index (FFMI).

**Results:**

DCAS ranged from −6:42 h to + 8:01 h and was not associated with body composition. Among adolescents with a later chronotype (*N* = 201) a 1 h increase in DCAS (later consumption of ‘highest caloric intake’ in comparison to the median intake of that group), increased FFMI by 1.92 kg/m^2^ (95% CI: 0.15, 3.69, *p* value = 0.04) over a median follow-up of 0.94 year.

**Conclusion:**

Alignment of energy intake with individual chronotype appears beneficial for FFMI among those with a late chronotype.

## Introduction

Chrono-nutrition is a novel research field focusing on three dimensions: Timing, frequency, and regularity of dietary intake [1]. We will investigate the first dimension of this trio which has rarely been studied in adolescents [2]. Those aged 9–16 years constitute a vulnerable group for the development of increasing body weight and unfavorable changes of other body composition measures, e.g., Fat Mass Index (FMI), likely tracking into adulthood [3–5].

Interest in the timing of energy intake was first sparked by studies in rodents indicating particularly detrimental cardio-metabolic effects when energy intake was scheduled to occur before or during the inactive period, i.e., periods corresponding to the evening in humans. Correcting the timing of food intake could prevent such effects [6, 7]. Subsequently, studies among humans followed with the perspective on the optimal timing of caloric intake. Observational studies linked the preferred consumption of habitual energy intake in the evening to higher total energy intake also during adolescence [8–10]. In consequence, higher evening energy intake is discussed to predispose to increases in BMI among adolescents [11].

Taken together, current evidence—albeit preliminary—suggests that substantial health benefits, e.g., lower BMI [12], which is an important risk factor for many chronic diseases, such as diabetes, cardiovascular diseases, or cancer, may arise from the consumption of energy at earlier times of the day. However, recent studies that address food timing in the context of the personal chronotype, i.e., individual preference in sleep timing, challenge this hypothesis [12, 13]. While emerging evidence suggests that those with a later chronotype are at higher risk for weight gain [14] and higher BMI [15, 16], shifting energy intake to earlier times of the day, against their underlying chronotype, may not be associated with the same benefit as seen among persons with an earlier chronotype. One explanation could be that timing of food intake, which diverges from the individual chronotype, constitutes a characteristic of a mismatch between the inner clock, i.e., chronotype, and the outer clock, i.e., timing of energy intake. On the contrary, alignment between eating time in accordance to ones chronotype may influence metabolic health beneficially [13]. Of note, chronotype itself is determined by genetics, age, sex, and environment [17]. During adolescence, a shift from an earlier to a later chronotype occurs naturally. Around the age of 20 years, the chronotype is most delayed and then tends to become earlier again [18].

Thus far, no studies have addressed whether timing of energy intake in accordance with the biological clock (i.e., early vs. late chronotype) is associated with beneficial body compositional developments during adolescence (age 9–16 years). For this purpose, we defined alignment as the earlier timing of ‘highest caloric intake’ per individual in comparison to the median timing of ‘highest caloric intake’ among persons with an earlier chronotype and vice versa among persons with a later chronotype. We defined ‘highest caloric intake’ as consuming at least 20% of total daily energy intake that is 10% above the definition for a ‘meal’ [9, 10].

We hypothesized that alignment between the circadian timing of ‘highest caloric intake’ and the individual chronotype among adolescents is associated with decreases in Body Mass Index-(Standard deviation score) [BMI (-SDS)] and FMI and/or increases in Fat Free Mass Index (FFMI).

## Materials and methods

### Study design

The DONALD study is an ongoing, prospectively designed open cohort study conducted in Dortmund, Germany. Since 1985, data on diet, growth, and developmental and metabolic factors are collected continuously from infancy (age 3 months) to adulthood. Approximately 30–35 healthy infants from Dortmund and surrounding communities, whose mothers and/or fathers have a sufficient level of the German language, are recruited every year via personal contacts, maternity wards, or pediatric clinics. The examination schedule includes quarter-yearly examinations in infancy, half-yearly examinations in the second year of life, and annual examinations thereafter until young adulthood. Among others, examinations include anthropometric measurements, lifestyle questionnaires, and a 3-day food record. Chronotype assessment in the DONALD study started in 2014 for participants from 9 years onwards by use of the Munich Chronotype Questionnaire (MCTQ) [17, 18]. Detailed information regarding the study design can be found elsewhere [19].

### Study sample

Until July 2020, *n* = 652 study participants completed the MCTQ (*N* = 1.237 Q). Questionnaires collected during the first 2 weeks after the time change in Germany from standard winter to summer time or vice versa [10] (*N* = 95) and questionnaires with missing values (*N* = 38) were excluded. The sample was further reduced due to missing information on in parallel collected dietary data leaving a total number of *n* = 259 participants (*N* = 461 questionnaires). Of those, (*n* = 196 out of 172 families) were adolescent (aged 9–16 years) [20] and provided *N* = 401 questionnaires (Appendix Fig. 3).

### Dietary assessment

Dietary intake in the DONALD study is assessed by use of 3-day weighed dietary records. The participants are free to choose the consecutive days, meaning that week and weekend days can be recorded. All foods and beverages consumed, as well as leftovers, are weighed and recorded over three consecutive days by the parents or by the older participants themselves with the use of regularly calibrated electronic food scales [initially Soehnle Digita 8000 (Leifheit AG, Nassau,Germany), now WEDO digi 2000 (Werner Dorsch GmbH, Muenster/Dieburg, Germany)]. When exact weighing is not possible, household measures (e.g., spoons and cups) are allowed for semi-quantitative recording. Information on recipes and on the types and brands of food items consumed is also requested. Additionally, participants record the time of every eating occasion. Energy and macronutrient intakes were calculated using the continuously updated in-house nutrient database LEBTAB [21], which is based on German standard food composition tables. Energy and nutrient contents of commercial food products are calculated by recipe simulation using labeled nutrient contents and ingredients. Macronutrients were considered as percentages of total energy intake (TEI). Subsequently, the individual means of TEI and macronutrient intakes were calculated from the three record days. The validity of dietary recording was previously evaluated by Bokhof et al. [22].

### Chronotype

The MCTQ [17, 18] includes questions regarding sleep and wake times during the week and weekend. The individual chronotype (continuously in h:min) was calculated as the Midpoint of Sleep, i.e., the half-way point between sleep-onset and sleep-end on free days (MSF) [17]. If applicable, the MSF is corrected for “oversleep” on free days to account for sleep-debt accumulated over the week (MSFsc) [18].

**Fig. 1:**
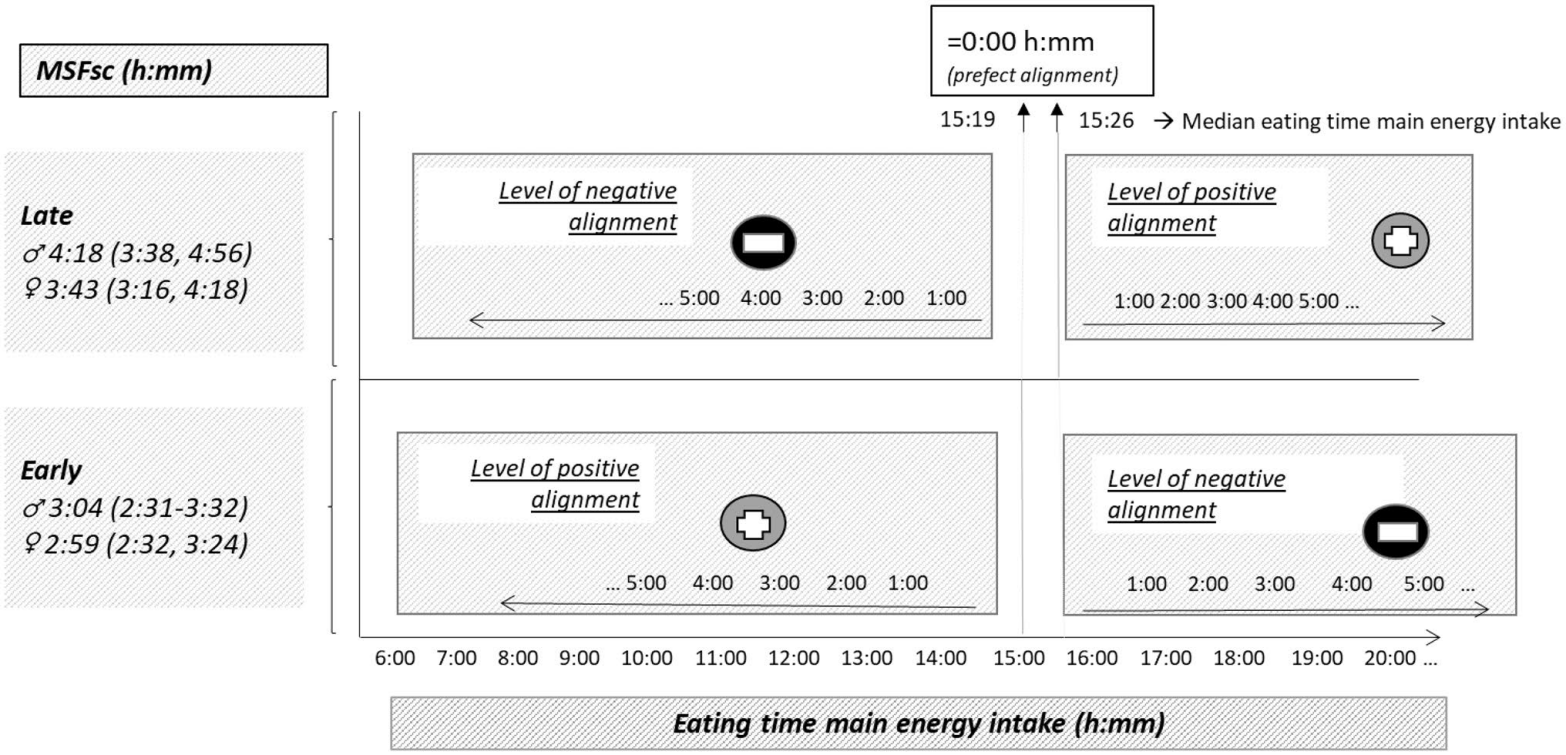
Diet-chrono alignment scoring (DCAS). *MSFsc* Midpoint of sleep (chronotype) corrected for sleep-debt accumulated over the workweek; *h* hours; *m* minutes. Alignment between chronotype and time of ‘highest caloric intake’ was expressed as the number of hours diverging from the chronotype-specific median eating time of ‘highest caloric intake’. Diet data were assessed by 3-day weighed dietary records, from which we derived average timing of ‘highest caloric intake’. Earlier eating resulted in positive divergence from the chronotype-specific reference eating time (defined as the median eating time of the specific chronotype), whereas later eating resulted in negative divergence from the reference for early chronotypes and vice versa for late chronotypes. MSFsc was regressed on age and sex. We ranked the residuals by the group of two and show in this figure the sex-specific MSFsc hours for early and late chronotypes

Boys and girls are different in their individual level of lateness; girls tend to be earlier in comparison to boys [23]. To account for age and sex differences in chronotype, we derived MSFsc residuals for all observations independent of age and sex and ranked them by the group of two (PROC RANK in SAS®). Afterward, we calculated median MSFsc hours stratified by sex for adolescents with earlier and later chronotype (Fig. 1).

### Diet-chrono-alignment score (DCAS)

In a first step, we calculated the sum of energy intake per eating occasion, e.g., all foods and beverages consumed within a 30-min time period were summarized into one eating occasion; and all eating occasions < 10 kcal were added to the previous eating occasion [10]. From this, we derived the time of the day at which the greatest amount of energy (> 20% of total daily energy intake) was consumed (timing of ‘highest caloric intake’ per day). Then, these individual eating times were averaged and defined as ‘highest caloric intake’ derived from 3-day dietary records.

In a second step, age- and sex-adjusted median eating times, for which we applied the residual method, were derived for adolescents with earlier and later chronotype. The median eating time was defined based on the sample population and served as the reference point for the definition of diet-chrono-alignment. Third, individual diet-chrono-alignment score (DCAS) was calculated as the difference in hours between the chronotype-specific median eating time of ‘highest caloric intake’ and the individual eating time of ‘highest caloric intake’. Hence, earlier eating in comparison to the population median eating time of ‘highest caloric intake’ resulted in positive alignment for adolescents with an earlier chronotype measured in hours, whereas for adolescents with a later chronotype, later eating resulted in positive alignment (Fig. 1).

### Anthropometric measures

Adolescents were measured annually by trained nurses according to standard procedures, dressed in underwear and barefoot. Standing height is measured to the nearest 0.1 cm using a digital stadiometer. Weight was measured to the nearest 0.1 kg with an electronic scale (model 753 E; Seca, Hamburg, German). Skinfold thickness was measured on the right side of the body at the biceps, triceps, subscapular, and suprailiac sites to the nearest 0.1 mm with a Holtain caliper (Holtain Ltd., Crymych, UK) [19]. Sex- and age-independent body mass index (BMI kg/m^2^) standard deviation scores (SDS) were calculated using the German national reference data according to the LMS Method [24]. Percent body fat (%BF) was estimated from two skinfolds (triceps, subscapular) using age-specific Slaughter equations [25], for the subsequent calculation of FMI (fat mass/m^2^; where fat mass = body weight * BF%/100) and FFMI (fat free mass/m^2^; where fat free mass = body weight − fat mass). Since the distribution of FMI was skewed, log10-transformed values were used in the analyses.

### Assessment of potential covariates

Parents are interviewed regarding family characteristics (i.e., parental education, smoking, and persons in the household). Every 4 years, maternal body weight and height were measured with the same equipment as for the children on the child’s admission to the study center.

Adolescents’ pubertal status was defined in accordance with the onset and the end of pubertal growth spurt. Age at Take-Off (ATO) is the age of minimal growth velocity. Age at Peak Height Velocity (APHV) is the age of maximal height velocity. ATO and APHV were derived from the parametric Preece and Baines Model 1 [26], details are explained elsewhere [26, 27]. Under- and over-reporting of dietary intake was assigned if TEI was unrealistic in relation to the estimated basal metabolic rate (according to age- and sex-specific equations of Schofield [28]). Based on the pediatric cut-offs by Sichert-Hellert et al. [29] we detected 64 under-reporters (15.9%) and no over-reporting of dietary records.

### Statistical analyses

All statistical analyses of the present evaluation were performed using SAS® procedures (version 9.4; Cary, NC, USA). The significance level was set at *p* < 0.05.

Linear mixed-effects regression models (PROC MIXED in SAS), including both fixed and random effects accounting for the nested nature of our data (children within families in the random statement) and the lack of independence between repeated observations on the same person. A further advantage is that the inclusion of all measurements is possible also in case of missing data for a specific point in time [30].

Repeated-measures regression models (PROC MIXED) were used to examine change-on-change associations of (a) the cross-sectional and (b) the longitudinal associations between the DCAS at first assessment and (Δ) body composition as well as (c) the respective changes in associations of (Δ) in DCAS and (Δ) BMI(-SDS), (Δ) FMI, and (Δ) FFMI over time [30, 31]. Variables of change were calculated by subtracting the baseline value from value at each year of assessment with 0 difference at first assessment.

Covariates for model adjustment were selected according to known predictors of BMI, body composition, and timing of energy intake [16, 32, 33]. From here, we identified minimally sufficient adjustment sets (msas) using a diagram (Appendix Fig. 4) representing the relationships among the identified variables [34]. Besides age at baseline and time between first and subsequent measurements (basic model) [31], sex, energy intake (g/d), physical activity (high vs. low), age at take-off and age at peak height velocity as puberty markers, smoking in the household, social jetlag, season of record (spring/summer/autumn/winter), number of questionnaires, and underreporting of dietary intake were important covariates.

Model building was driven by the log-likelihood criterion to define the final crude model. The Akaike Information Criterion (AIC) and the Bayesian Information were used to select the correlation structure best describing the correlated nature of the data. Tests for effect modification were performed by the inclusion of interaction terms between DCAS and MSFsc or sex.

To manage missing data, we undertook multiple imputations, using the MI procedure in SAS and explored the pattern of missingness. We generated an imputed database containing five imputed versions to predict missing values for ATO (*n* = 72, 18%) and physical activity (*n* = 67, 17%). Final models were tested regarding multicollinearity, heteroscedasticity, and normal distribution of residuals. Finally, we excluded participants who used an alarm clock during the weekend (*n* = 59, 15%) in a sensitivity analyses and assessed the influence of ‘eveningness in energy intake’ on body compositional measures. ‘Eveningness in energy intake’ was defined as percentage of energy intake in the evening (after 6 pm until 11:15 pm) − percentage of energy intake in the morning (before 11 am starting at 5:15 am) [9].

## Results

Sample characteristics of the first and last assessment, stratified by adolescents with an earlier and later chronotype, are presented in Table 1. The sample used for the analyses constituted 41% female participants (*N* = 81). All body compositional measures were slightly higher in those adolescents with a later chronotype. Mean values of MSFsc differed between adolescents with earlier and later chronotype by more than 1 h for the first and last measurement. While sleep duration by chronotype was not different at the first measurement but differed with almost 0:30 min at the last measurement.

**Table 1:** Characteristics of 196 adolescents (age 9–16 years) who provided 401 MCTQ questionnaires stratified by adolescents with an earlier (*n* = 86) and later (*n* = 110) chronotype (MSFsc), The DONALD Study, 2015–2020^a^

	Chronotype			
	First assessment		Last assessment	
	Early	Late	Early	Late
Female (*n* (%))	30 (15)	51 (26)	40 (20)	41 (21)
Follow-up (years)	–	–	1.85 ± 1.55	1.30 ± 1.51
Number of questionnaires	1 (1, 1)	1 (1, 1)	2 (1, 3)	2 (1, 3)
Age (years)	12.19 ± 1.81	12.26 ± 1.74	13.94 ± 1.70	13.65 ± 1.67
Puberty				
*Boys*				
ATO	10.26 ± 1.70	10.28 ± 1.46		
APHV	12.91 ± 2.42	12.71 ± 2.00		
*Girls*				
ATO	9.65 ± 1.75	8.76 ± 1.55		
APHV	12.80 ± 2.57	11.26 ± 1.86		
Anthropometric data				
BMI (kg/m^2^)	18.56 ± 4.47	18.88 ± 3.13	19.76 ± 4.41	19.81 ± 3.32
BMI-SDS (kg/m^2^)	−0.17 ± 1.17	0.03 ± 0.94	−0.12 ± 1.15	0.05 ± 1.00
FMI (kg/m^2^)	2.95 (1.98, 4.22)	3.27 (2.41, 4.72)	3.26 (2.08, 5.21)	3.58 (2.67, 4.86)
FFMI (kg/m^2^)	14.55 ± 1.65	14.97 ± 1.73	15.27 ± 1.66	15.56 ± 1.87
Chronobiological variables (h:mm)				
MSFsc	2:49 ± 0:40	3:57 ± 0:51	3:15 ± 0:38	4:31 ± 0:57
Sleep duration	8:53 ± 1:00	8:52 ± 0:52	8:09 ± 1:00	8:26 ± 0:57
Using an alarm during the weekend	7 (8)	12 (11)	10 (10)	11 (11)
Spring	17 (9)	18 (9)	19 (10)	17 (9)
Summer	22 (11)	37 (19)	27 (14)	36 (18)
Autumn	22 (11)	23 (11)	27 (14)	15 (8)
Winter	25 (13)	32 (16)	26 (13)	29 (15)
Other risk factors				
Low physical activity (kcal/d)^b^	38 (19)	56 (29)	45 (23)	45 (23)
Parental characteristics				
Maternal education (high)^c^	77 (39)	91 (46)	89 (45)	79 (40)
Maternal employment (yes)	80 (41)	101 (52)	92 (47)	89 (45)
Maternal overweight (yes)^d^	37 (19)	46 (32)	38 (19)	45 (23)
Smoking in the household (yes)	6 (3)	17 (9)	7 (4)	15 (8)

*MCTQ* Munich ChronoType Questionnaire; *MSFsc* midpoint of sleep (chronotype) corrected for sleep-debt accumulated over the workweek; *BMI* body mass index; *SDS* standard deviation score; *FMI* fat mass index; *FFMI* fat free mass index

^a^Results are presented as number and (percentage) or mean ± standard deviation

^b^Expressed as energy expenditure in kcal based on the sum of organized and unorganized sport activities

^c^ ≥ 12 years of schooling

^d^ ≥ 25 kg/m^2^

Dietary characteristics of the first and last assessment, stratified by adolescents with an earlier and later chronotype, are presented in Table 2. The DCAS ranged from −6:42 to + 8:01 h. While adolescents with a later chronotype at first assessment showed negative alignment of 7 min (earlier eating), they adapted toward later eating until last assessment. Macronutrient intake was not different by chronotype. In contrast to those having a late chronotype, adolescents with an earlier chronotype are more likely to have breakfast (93% vs. 96% having breakfast, considering the last assessment). No one with a later chronotype skipped dinner.

**Table 2:** Dietary characteristics of 196 adolescents (age 9–16 years) who provided 401 dietary records stratified by adolescents with an earlier (*n* = 86) and later (*n* = 110) chronotype (MSFsc), DONALD Study, 2015–2020^a^

	Chronotype			
	First assessment		Last assessment	
	Early	Late	Early	Late
DCAS (min)	00:19 (−0:01, 2:05)	−0:07 (−1:18, 1:38)	0:02 (−1:38, 1:42)	0:23 (−1:07, 1:53)
Time of ‘highest caloric intake’ (h:mm)	14:47 ± 2:26	15:32 ± 2:18	15:23 ± 2:15	15:41 ± 2:27
Dietary intake over the whole day				
TEI (kcal/d)	1880 (1625, 2088)	1833 (1575, 2112)	1950 (1629, 2288)	1932 (1672, 2289)
Fat (%TEI)	34 ± 6	35 ± 6	35 ± 6	36 ± 6
Carbohydrates (%TEI)	51 ± 6	51 ± 6	51 ± 6	51 ± 6
Protein (%TEI)	13 ± 3	13 ± 2	14 ± 3	14 ± 2
Dietary intake for the eating occasion with ‘highest caloric intake’				
TEI (kcal)	689 ± 27	654 ± 25	724 ± 34	731 ± 27
%TEI	37 ± 7	35 ± 6	37 ± 7	37 ± 6
Carbohydrates (%E)	47 ± 8	47 ± 9	45 ± 10	45 ± 11
Protein (%E)	14 ± 3	14 ± 4	15 ± 4	14 ± 4
Fat (%E)	38 ± 8	38 ± 9	39 ± 9	39 ± 10
Dietary intake morning before 11 am				
%TEI	27 ± 11	23 ± 9	24 ± 9	22 ± 11
Daytime dietary intake 11 am to 6 pm				
%TEI	43 ± 11	43 ± 10	43 ± 12	43 ± 11
Dietary intake evening after 6 pm				
%TEI	28 ± 11	32 ± 9	31 ± 12	33 ± 9
Breakfast skipping^b^				
Yes	5 (3)	8 (4)	8 (4)	14 (7)
Lunch skipping^c^				
Yes	0 (0)	0 (0)	1 (1)	0 (0)
Dinner skipping^d^				
Yes	3 (3)	0 (0)	3 (3)	0 (0)
No weekdays per diet records^e^				
1	39 (20)	45 (23)	47 (24)	47 (24)
2	18 (9)	24 (12)	18 (9)	18 (9)
3	29 (15)	41 (21)	34 (17)	34 (17)

*DCAS* diet-chrono-alignment score; *%TEI* percentage of total daily energy intake; *%E* energy percentage

^a^Results are presented as medians (q1: q3), mean ± standard deviation or number and (percentage)

^b^Defined as having consumed no energy intake before 11 am on at least two of three record days

^c^Defined as having consumed no energy intake between 11 am and 6 pm on at least two of three record days

^d^Defined as having consumed no energy intake after 6 pm on at least two of three record days

^e^1 = 2 days of diet recording during the weekend (WE), 2 = 1 day during the WE, 3 = no records during the WE

**Fig. 2:**
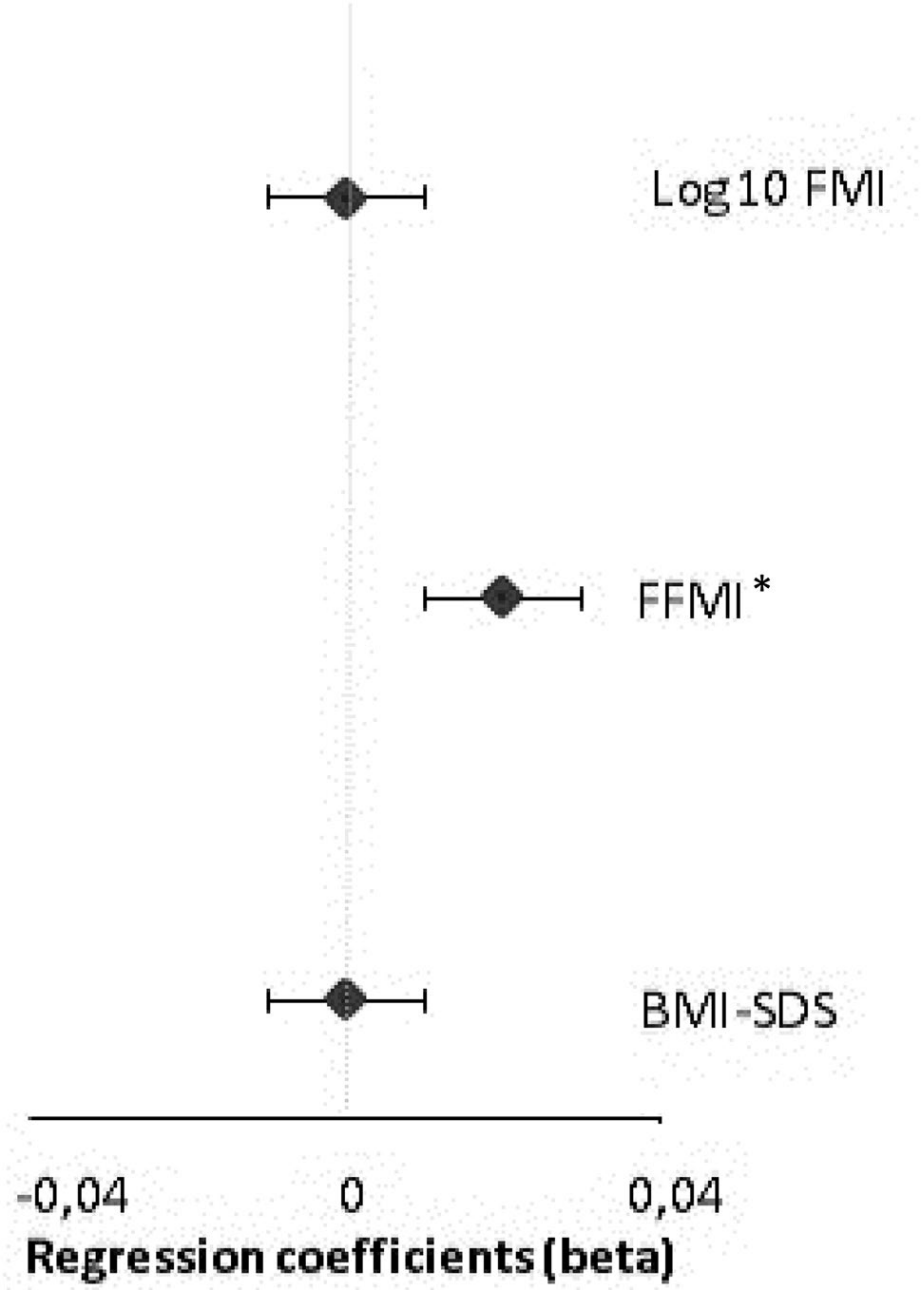
Regression coefficients and 95% confidence intervals^a,b^ of the longitudinal association (model b) between ‘eveningness in energy intake’ and body composition in later chronotypes (*N* = 201 questionnaires, *n* = 110 participants). *FFMI* Fat Free Mass Index (kg/m^2^); *FMI* Fat Mass Index (kg/m^2^); *BMI-SDS* Body Mass Index-Standard Deviation Score (kg/m^2^). *Statistically significant *p* value < 0.05, per 1% more energy intake in the evening FFMI increase by 0.02 kg/m^2^ in late chronotypes. ^a^Models contain a random intercept with an unstructured covariance (UN). ^b^All models were adjusted for energy intake (g/day), number of questionnaires, physical activity level (high/low, based on the median), age at take-off and age at peak height velocity as puberty markers, season of record (spring/summer/autumn/winter), smoking in the household, social jetlag, and underreporting of energy intake

Table 3 shows no associations of DCAS with BMI-SDS or FMI in any of the adjusted models (a–c), neither in the total sample nor stratified by adolescents with an earlier and later chronotype (interactions between DCAS and chronotype: for BMI-SDS, *p* value < 0.05, for FMI *p* value > 0.05). Whereas there was a positive longitudinal association between DCAS and FFMI development in those adolescents with a later chronotype (model b). A 1 h increase in later eating, compared to the median intake time of late chronotypes, resulted in a longitudinal increase of ∆ FFMI by 1.92 kg/m^2^ (*p* = 0.04) over time (model b, Table 3). We found no significant interaction by sex. The sensitivity analyses in which we excluded participants who used an alarm clock during the weekend yielded similar results (data not shown). Figure 2 visualizes the longitudinal relationships between ‘eveningness in energy intake’ and long-term ∆ BMI-SDS, ∆ FFMI, and ∆ FMI (model b) for adolescents with a later chronotype. A 10% larger energy intake in the evening rather than in the morning increased FFMI prospectively by 0.2 kg/m^2^ (95% CI: 0.1, 0.3).

**Table 3:** Associations between the Diet-Chrono-Alignment Score (DCAS) and BMI-SDS, FFMI, or FMI during adolescence [regression coefficients (ß) with 95% confidence intervals (CI)] by early and late chronotype groups: The DONALD-study (*N* = 401 questionnaires of *n* = 196 participants)

		Crude model		Final adjusted model	
	*N* (*n*)	ß and (CI)	*p*	ß and (CI)	*p*
BMI					
*Cross-sectional association between dietary alignment and BMI-SDS (model a)*					
Overall	401 (196)	0.77 (−0.74, 2.27)	0.31	0.72 (−0.74, 2.18)	0.33
Early chronotype	200 (86)	0.57 (−1.66, 2.80)	0.62	0.60 (−1.72, 2.92)	0.59
Late chronotype	201 (110)	1.01 (−0.91, 2.93)	0.28	0.85 (−1.05, 2.75)	0.34
*p value* for interaction* = 0.01					
*Longitudinal association between dietary alignment and BMI-SDS (model b)*					
Overall	401 (196)	0.16 (−0.24, 0.56)	0.43	0.06 (−0.34, 0.47)	0.76
Early chronotype	200 (86)	0.02 (−0.54, 0.58)	0.94	−0.02 (−0.60, 0.57)	0.96
Late chronotype	201 (110)	0.81 (0.09, 1.53)	0.03	0.48 (−0.36, 1.32)	0.23
*p value* for interaction* = 0.001					
*Association between change in alignment and change in BMI-SDS (model c)*					
Overall	401 (196)	1.12 (−0.31, 0.56)	0.57	0.09 (−0.36, 0.53)	0.69
Early chronotype	200 (86)	0.34 (−0.31, 0.99)	0.29	0.37 (−0.26, 0.99)	0.23
Late chronotype	201 (110)	0.41 (−0.33, 1.14)	0.26	−0.04 (−0.85, 1.32)	0.92
*p value* for interaction* = 0.03					
FFMI					
*Cross-sectional association between dietary alignment and FFMI (model a)*					
Overall	401 (196)	0.54 (−1.68, 2.75)	0.63	0.69 (−1.47, 2.85)	0.52
Early chronotype	200 (86)	1.58 (−1.47, 4.63)	0.30	1.69 (−1.43, 4.82)	0.27
Late chronotype	201 (110)	−0.81 (−3.89, 2.28)	0.59	−1.07 (−4.13, 1.99)	0.45
*p value* for interaction* = 0.47					
*Longitudinal association between dietary alignment and FFMI (model b)*					
Overall	401 (196)	0.17 (−0.66, 0.99)	0.69	0.21 (−0.60, 1.01)	0.61
Early chronotype	200 (86)	−0.40 (−1.38, 0.58)	0.41	−0.33 (−1.30, 0.64)	0.36
Late chronotype	201 (110)	1.56 (−0.09, 3.20)	0.06	1.92 (0.15, 3.69)	0.04
*p value* for interaction* = 0.03					
*Association between change in alignment and change in FFMI (model c)*					
Overall	401 (196)	0.00 (−0.84, 0.85)	0.99	0.11 (−0.73, 0.95)	0.80
Early chronotype	200 (86)	−0.31 (−1.58, 0.96)	0.62	−0.06 (−1.15, 1.03)	0.97
Late chronotype	201 (110)	0.67 (−0.84, 2.18)	0.36	0.28 (−1.23, 1.80)	0.66
*p value* for interaction* = 0.69					
Log^10^FMI					
*Cross-sectional association between dietary alignment and FMI (model a)*					
Overall	401 (196)	0.28 (−0.48, 1.04)	0.47	0.26 (−0.47; 0.99)	0.48
Early chronotype	200 (86)	−0.08 (−1.22, 1.05)	0.88	−0.05 (−1.19, 1.10)	0.93
Late chronotype	201 (110)	0.70 (−0.24, 1.69)	0.14	0.64 (−0.31, 1.58)	0.16
*p value* for interaction* = 0.17					
*Longitudinal association between dietary alignment and FMI (model b*					
Overall	401 (196)	0.03 (−0.21, 0.28)	0.78	−0.01 (−0.26; 0.24)	0.92
Early chronotype	200 (86)	0.11 (−0.26, 0.47)	0.55	0.13 (−0.23, 0.49)	0.46
Late chronotype	201 (110)	0.17 (−0.28, 0.58)	0.40	−0.06 (−0.56, 0.44)	0.80
*p value* for interaction* = 0.35					
*Association between change in alignment and change in FMI (model c)*					
Overall	401 (196)	0.14 (−0.12, 0.39)	0.29	0.10 (−0.16, 0.37)	0.43
Early chronotype	200 (86)	0.20 (−0.21, 0.60)	0.33	0.21 (−0.22, 0.65)	0.31
Late chronotype	201 (110)	0.31 (−0.17, 0.79)	0.20	0.05 (−0.46, 0.56)	0.83
*p value* for interaction* = 0.61					

*DCAS* Diet-Chrono-Alignment Score, based on early and late chronotypes, as well as early and late eating. Definitions for categorization were made independent of age and sex using the residual method. *N* number of questionnaires and dietary records; *n* number of participants; *BMI* Body Mass Index (kg/m^2^); *FFMI* Fat Free Mass Index (kg/m^2^)

^*^p value for interaction between chronotype and the DCAS

^a^Models contain a random intercept and slope for time with an unstructured covariance (UN). *Crude model*: Basic model containing age at baseline and time between last and first measurement. *Final adjusted model*: Crude model additionally adjusted for energy intake (g/day), number of questionnaires, physical activity level (high/low, based on the median), age at take-off and age at peak height velocity as puberty markers, season of record (spring/summer/autumn/winter), smoking in the household, social jetlag, and underreporting of energy intake

## Discussion

In this longitudinal designed cohort study, we examined the cross-sectional, longitudinal, and change-on-change associations between alignment in dietary energy intake timing according to chronotype in relation to BMI (-SDS), FMI, and FFMI during adolescence. We found no overall associations between the DCAS and anthropometrics. However, those adolescents who had a late chronotype and ate their meal with ‘highest caloric intake’ later in the day, in comparison to the median specific eating time in that group, showed an increase in FFMI over time. To the best of our knowledge, this is the first study that has defined a score representing the alignment between dietary intake timing and chronotype.

### Later and earlier eating

Studying BMI as the outcome of interest should be accompanied by additional measures on body composition, such as FMI or FFMI, especially during adolescence [35]. However, most previous studies did not consider body compositional measures next to BMI, which limits comparability with previous research. Nevertheless, Eng et al. [36] reported a paradox in their analyses of the National Health and Nutrition Examination Survey (NHANES) 1999–2004, *N* = 11,072 aged 2–18 years. In children aged 2–11 years, later eating in the time between 4 pm to midnight was associated with increasing overweight defined as a BMI between the 85th and 94th percentile, and obese (≥ 95th percentile) applying Centers for Disease Control and Prevention (CDC's) BMI-for-age and sex-specific growth charts. Similar results as shown by Eng et al. for children below the age of 11 years were presented by Martínez-Lozano et al. [37] for the age 8–12 years. However, the opposite was true in NHANES for adolescents aged 12–18 years. Late-eating adolescents had 16 times lower odds for being overweight (ß: −15.9, *p* value = 0.01). This difference may partly be attributable to chronobiological changes toward a later chronotype starting around the age of 12 years [38]. In line with this, Munoz et al. [13] reported that also adults aged 18–65 years having a late chronotype and a higher %TEI at dinner were normal weight, whereas evening types who had more %TEI in the morning were more likely to be overweight. Coulthard et al. [39] found no association between eating after 8 pm and excessed weight or increased energy intake in 1.620 UK children aged 4–10 years. Also, no effect was observed in the CHOP trial where they included 729 healthy children to investigate the distribution of eating occasions on *z*BMI [40]. These findings support our null findings for BMI-SDS.

### Eveningness in energy intake

Results of the CRO-PALS Longitudinal Study including 607 adolescents showed no associations between ‘eveningness in energy intake’, based on a single 24-h recall and BMI or waist–hip-ratio [41]. This is in line with our results on ‘eveningness in energy intake’ and BMI-SDS. The association between ‘eveningness in energy intake’ and FFMI supported our results of the main analyses. The weaker association of our sensitivity analyses for FFMI may suggest that not solely the amount of energy intake eaten earlier or later during the day, but especially the balance with the underlying chronotype, hence the internal clock, may be of particular interest regarding beneficial longitudinal developments in body composition. The importance of dietary intake timing as an independent risk factor besides the amount of food intake is supported by an intervention trial based on 110 participants aged 18–22 years in which timing of food consumption relative to clock hour and endogenous circadian time was assessed in relation to body composition [42].

### Biological background

The timing of food intake is important in harmonizing the central and peripheral clocks. A desynchrony between both may lead to metabolic distortion and unbeneficial body compositional developments [43]. Earlier, we have shown in DONALD that children, while they age, do change the amount of energy intake to later times of the day [9]. Such an observation requires a supportive family environment enabling later eating times. In line with this, Morgan et al. [44] reported earlier on the benefits in autonomous decisions by emerging adults regarding eating regulation styles by parents on body fat. The adaptation toward later eating hours in adolescents who naturally develop toward later chronotypes may contribute to a better alignment between their internal and external clock. The benefits of such an alignment has been elaborated on in the other studies [13, 43] and is supported by our findings.

To summarize, a large body of evidence suggests ‘later’ eating as being unbeneficial for metabolic health [42]. Especially, in adults. However, this does not seem to be applicable for everybody [13, 43]. In the current study, we could show that the best time for food consumption depends on a person’s individual chronotype. Not considering chronotype in the analyses may partly explain the large controversy we find in the current literature [45, 46]. Another possible reason for the discrepancies in results between ‘later’ energy intake and body composition could be related to the definition of eating time. For instance, ‘late’ energy intake 2 h before bedtime [12] or closer to melatonin onset (i.e., biological night) [42] represents more extreme ‘lateness’ and shows positive associations with overweight in adult later chronotypes.

### Strength and limitations

A major advantage of the DONALD Study is the longitudinal study design, enabling the examination of longitudinal associations. Other studies had to rely on cross-sectional data mainly [39]. Further strengths are the dietary assessment method, avoiding recall bias, and the assessment of chronotype using the validated MCTQ [18, 47] which was applied previously among adolescents in the other studies [23, 48]. In addition, the consideration of sleep/wake cycles which are used for chronotype development were shown earlier to be a practical approach for the definition of circadian timing of food intake [12, 42].

Generalizability of our results may be limited, since the DONALD study is characterized by a homogeneous sample of German participants, represented by a high socioeconomic status (SES) [19]. Also, females were slightly underrepresented in the current analyses. Regarding body compositional measures, we would like to add that despite high SES of the DONALD population, the prevalence of overweight (15.5%) is very well comparable to the overall German population (15.4%) [49]. Another disadvantage is the small sample size. However, by generating the DCAS, we saved an additional level of stratification by early and late timing of ‘highest caloric intake’.

### Public health impact and future directions

This is the first analysis performed in adolescence showing longer term consequences of circadian alignment with energy intake timing. Supporting adolescents in following their individual clock appears to be most beneficial for a healthy body compositional development [35]. General practitioners should prepare and explain parents the natural occurring changes in chronobiology of their children they can expect during adolescence and enable parents to accept changes in timing of sleep-onset [18] as well as food intake [9]. Of note, it is not the later chronotype that increase the risk for future metabolic disease development, but the attached individual behavior [43]. The application of our approach to a larger and more heterogeneous sample of adolescence, that may also include participants not enabled by their parents to follow their internal clock, would enhance this field of research.

In conclusion, changes in chronobiology and eating time accompany the phase of the transition from childhood to adulthood. Chronotype as well as eating time advance naturally to later times of the day during adolescence. The alignment between dietary intake timing and chronotype appears to encourage favorable developments in body composition in adolescents with a later chronotype.

## Figures and Tables

**Fig. 3:**
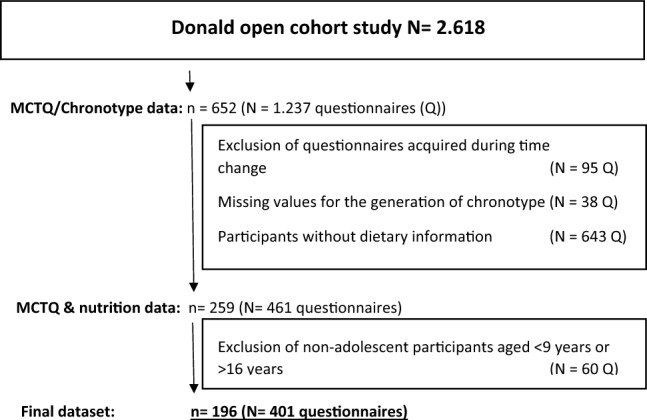
Flow diagram of the DONALD study sample for the selection of participants regarding the association between DCAS and body composition, July 2020. *DONALD* DOrtmund Nutritional and Anthropometric Longitudinally Designed; *MCTQ* Munich Chronotype QuestioNnaire; *DCAS* Diet-chrono-alignment score

**Fig. 4:**
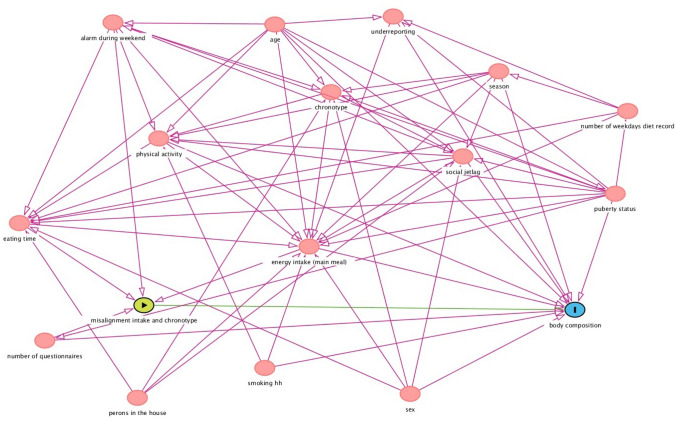
Directed Acyclic Graph^a^ for the association between the DCAS and body composition in adolescents. The minimally sufficient adjustment set revealed the following covariates: age at take-off and age at peak height velocity (puberty markers), age, energy intake (main meal), count of questionnaires, puberty status, season, sex, smoking in the household, social jetlag, and underreporting of dietary intake. ^a^Schipf S, Knüppel S, Hardt J, Stang A. Directed Acyclic Graphs (DAGs)—Die Anwendung kausaler Graphen in der Epidemiologie [Directed acyclic graphs (DAGs)—the application of causal diagrams in epidemiology]. Gesundheitswesen. 2011 Dec; 73(12):888–92. German. https://doi.org/10.1055/s-0031-1291192. Epub 2011 Dec 22. PMID: 22193898

## Data Availability

Data of the DONALD study is available upon request to epi@uni-bonn.de.

## Appendix

See Figs. 3 and 4.
